# Supplementary material for: Highly Specific Memory B Cells Generation after the 2nd Dose of BNT162b2 Vaccine Compensate for the Decline of Serum Antibodies and Absence of Mucosal IgA
Source: Cells. 2021 Sep 26;10(10):2541. doi: 10.3390/cells10102541 (PMC8533837; doi:10.3390/cells10102541)
Supplement: Supplementary file 1 [file cells-10-02541-s001.zip › cells-1374616-supplementary.pdf]

Supplementary Materials

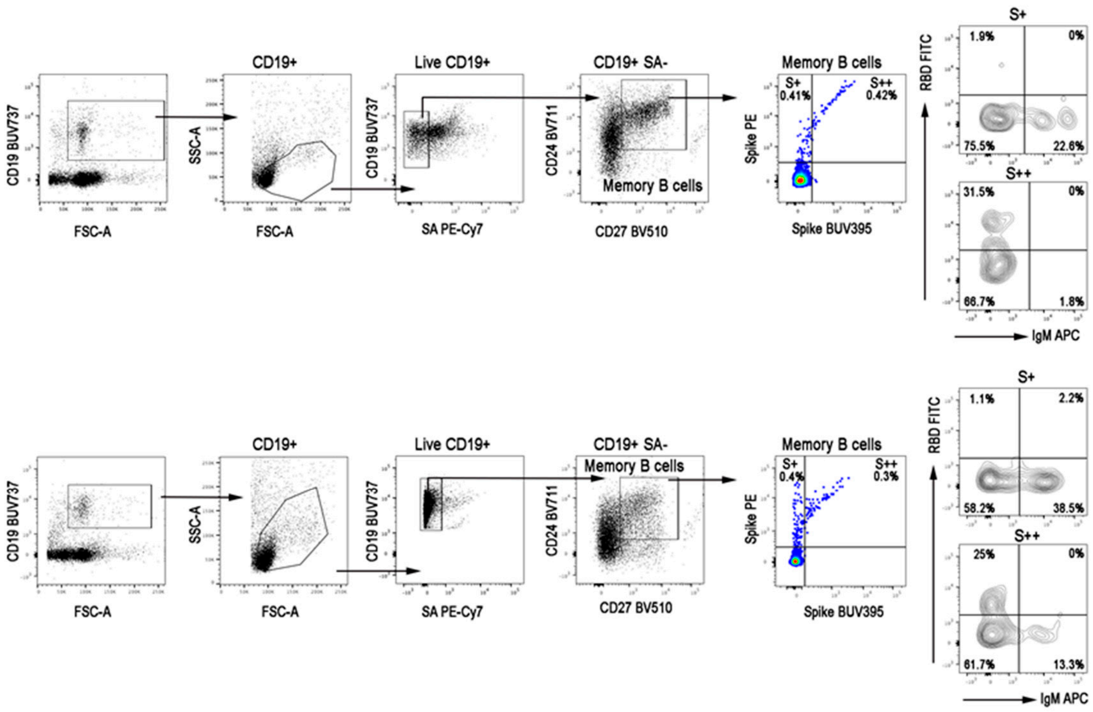

Figure S1: Spike-specific memory B cells in convalescent COVID-19 patients**.** Gating strategy to identify S+ and S++ MBCs. S+ MBCs were identified as CD19+PECy7-CD24+CD27+CD38-Spike-PE+SpikeBUV395-; S++ MBCs were gated as CD19+PECy7-CD24+CD27+CD38-Spike-PE+SpikeBUV395+. Identification of RBD+ cells inside S+ and S++ MBCs in two representative convalescent patients

Supplementary Table 1: Antibodies for staining.

	Clone.	Catalog number..
CD19 BUV737.	SJ25C1	612757
CD19 BV786.	SJ25C1	563325
CD24 BV711.	ML5	563401
CD27 BV510.	T-271	740167
CD38 BV421.	HIT2	562444
IgG BV650.	G18-145	740596
IgM APC	Polyclonal	709-136-073
Streptavidin PE.		554061
Streptavidin BUV395.		564176
Streptavidin FITC.		554060
Streptavidin PE-Cy7.		557598
